# Fundamental dermatology education of medical doctors at a regional hospital in Johannesburg, South Africa

**DOI:** 10.11604/pamj.2024.47.178.40779

**Published:** 2024-04-09

**Authors:** Tamara Romanini, Jedd Hart

**Affiliations:** 1 Emergency Department, Thelle Mogoerane Regional Hospital, Johannesburg, South Africa,; 2 Division of Emergency Medicine, Faculty of Health Sciences, University of the Witwatersrand, Johannesburg, South Africa

**Keywords:** Humans, dermatology, surveys and questionnaires, curriculum

## Abstract

**Introduction:**

dermatology is a prevalent field of global health and dermatological conditions are amongst the most frequent complaints affecting communities, yet dermatology has become an overlooked aspect of the medical school curricula and many medical schools have failed to provide doctors with training to manage these conditions confidently and adequately. This study aimed to determine the baseline dermatological knowledge of medical doctors and determine the influence of fundamental dermatology education on hospital medical staff at a regional hospital in Johannesburg, South Africa.

**Methods:**

the knowledge and confidence of 33 medical doctors were tested using a pre-test and post-test quasi-experimental design. Participants completed an online questionnaire followed by an image-based test consisting of 20 questions to determine their level of confidence in diagnosing and managing common dermatological conditions. The participants then attended a sixty-minute educational session based on common dermatological conditions. Following this, their level of confidence and knowledge on the subject was re-assessed using the same online test. Pre and post-intervention confidence and test scores were compared.

**Results:**

the pre-intervention test mean score was 67.11%. The post-intervention mean score was 92.50%. The difference between means (post-intervention - pre-intervention) ± SEM was 25.39 ± 4.81. The intervention significantly improved overall test scores (p-value < 0.0001). Many participants felt that their undergraduate training was insufficient in preparing them for the management of common dermatological conditions.

**Conclusion:**

the baseline knowledge and confidence of medical doctors in managing common dermatological conditions was poor and such educational interventions have significant value in improving the ability of medical doctors in managing common dermatological conditions. More time should be dedicated to dermatology training at an undergraduate level.

## Introduction

Dermatology is a prevalent and ever-expanding field of global health. Skin and subcutaneous conditions are extremely common and cause a great deal of psychosocial stress. Chronic dermatological pathologies pose a significant risk to a patient´s mental health and have been correlated with a poorer overall quality of life [1]. Skin conditions also often act as the preface for a more severe underlying systemic condition such as human immuno-deficiency virus (HIV), autoimmune diseases, and neglected diseases such as elephantiasis and other lymphedema-causing diseases [1,2], therefore, it is important for such conditions to be correctly identified and appropriately managed. In order to achieve this, it is important for doctors to have adequate knowledge about common dermatological conditions. However, teaching in medical schools has been found to be insufficient in training doctors to accurately diagnose and manage basic dermatological conditions [3]. Dermatology has become an overlooked aspect of the medical school curricula and many medical schools have failed to prepare doctors to treat basic dermatological conditions [4].

The study aimed to determine whether an educational intervention improved the knowledge and confidence of medical doctors in diagnosing and managing common dermatological conditions. With these insights, we hope to not only improve the level of confidence of medical doctors in managing dermatological conditions but also prove the necessity of improving dermatological education for all medical doctors.

## Methods

**Study design:** the research conducted employed a pre-test and post-test quasi-experimental design.

**Setting:** it was performed at the Thelle Mogoerane Regional Hospital, Johannesburg, South Africa. Ethics clearance and hospital permission were granted in June 2022. Recruitment took place in July 2022. Participants were exposed to the research and data was collected in July 2022. Data was subsequently analysed in August-September 2022.

**Participants:** all qualified medical doctors working at the hospital were invited to participate in the study.

### Variables

**Level of confidence:** this variable aimed to assess the level of confidence of medical staff in diagnosing and managing common dermatological conditions presenting at the hospital.

**Online questionnaire:** an online questionnaire was created using Google Forms to collect data on the participants' confidence levels. This consisted of a 20-question, image-centric, multiple-choice test that focused on the recognition, diagnosis, and management of ten common dermatological conditions. Each question carried a score of one, resulting in a total score out of twenty.

**Educational session:** a sixty-minute educational session was conducted after the completion of the online questionnaire. The session involved a PowerPoint presentation centered on common dermatological conditions. Due to COVID-19 precautions, an online lecture format was chosen.

**Post-intervention assessment:** following the educational session, participants' confidence levels in diagnosing and managing dermatological conditions were re-assessed. The same multiple-choice test and Google Forms questionnaire were used to evaluate and quantify the potential benefit of the intervention.

**Data sources/measurement:** an online questionnaire was generated using Google Forms to determine the level of confidence of medical staff in diagnosing and managing common dermatological conditions presented to the hospital. A 20-question image-centric multiple-choice test related to the recognition, diagnosis, and basic management of ten common dermatological conditions was completed by use of the Google Form questionnaire. Each question was worth one mark and the total was scored out of twenty. A sixty-minute educational session with a PowerPoint presentation based on common dermatological conditions was conducted directly afterward. For the sake of COVID-19 precautions, an online lecture format was used. Following the presentation, the participant´s level of confidence in diagnosing and managing dermatological conditions was re-assessed using the same multiple-choice test and Google Forms questionnaire to assess and quantify the potential benefit of the intervention. Quality control was achieved by making use of image-centric question formats eliminating the possibility for online searches.

**Bias:** the risk of selection bias was minimized by ensuring that the study was not limited to one medical department. If participants had varying levels of prior dermatological training or experience, it could confound the impact of the intervention on their confidence, although it is difficult to account for every individual participant´s background the study took place at a regional hospital and so no dermatologists were in the cohort. Attrition bias was avoided by ensuring all participants who participated were aware of their responsibilities and fully participated in the study.

**Study size:** sample size required was calculated retrospectively using G-power software for the difference between means of matched pairs. Effect size (dz) was determined to be 5.97, noncentrality parameter δ was 10.34, and critical t 2.92. Conclusively, an adequate sample size was in this study used for statistical confidence α = 0.05.

**Quantitative variables:** the quantitative variables collected in the study, such as the participants' confidence scores were summarized using descriptive statistics. Measures such as mean, standard deviation, and 95% confidence intervals were calculated to provide an overview of the central tendency and variability of the data.

To assess the impact of the educational session on participants' confidence levels, a comparison between pre-and post-intervention scores was conducted. The differences in mean were examined to evaluate the effectiveness of the intervention.

Subgroup analyses were performed. The data were stratified based on participants' career levels. This allowed for examining potential variations in the intervention's effects across different subgroups.

**Statistical methods:** data was exported from Google Forms onto a password-protected Microsoft Excel Spreadsheet. Statistical analysis was conducted using GraphPad Prism version 8.0.3 and RStudio using the DplyR and ggplot2 packages. Statistical analysis was conducted using a Wilcoxon matched-pairs signed-rank test, p-values < 0.05 were considered to be significant. Descriptive statistics were used to compare trends in participants´ level of confidence and perceptions pre and post-intervention.

**Ethical considerations:** approval was granted by the Thelle Mogoerane Regional Hospital CEO and management team. Ethics clearance was obtained from The University of the Witwatersrand Human Research Ethics Committee (Medical) (reference number: M220446). Written informed consent was obtained from participants and confidentiality and anonymity were achieved by making use of an assigned unique identifier code.

## Results

**Participants and descriptive data:** thirty-three medical doctors participated: 23 medical officers, 7 interns, 2 registrars, and 1 consultant. The mean age was 28.24 years, the proportion of females to males was 1.54, the mean years of practice was 5.18 (Table 1). The study had a low participation rate which could be because attendance at such sessions is difficult during working hours.

**Table 1:** summary of the key characteristics of the study population

Characteristic	Value
Mean age	28.24
Proportion of females: males	1.54
Mean years of practice	5.18
Proportion of interns	0.21
Proportion of medical officers	0.70
Proportion of registrars	0.06
Proportion of consultants	0.03

**Figure 1:**
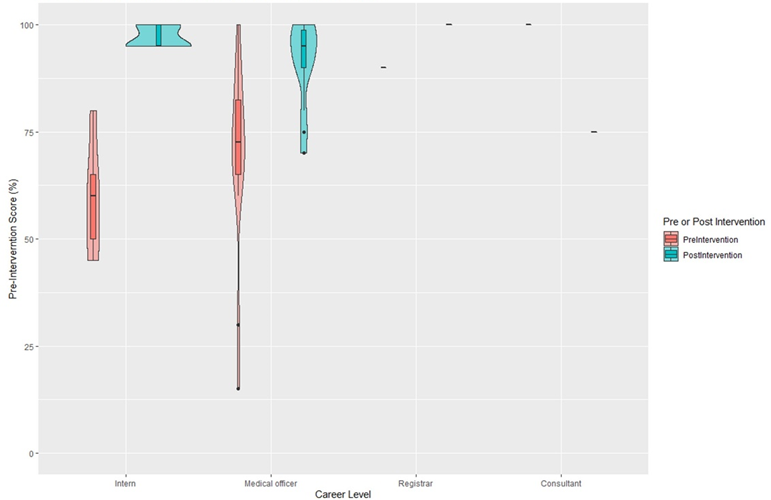
violin plot comparing pre-intervention and post-intervention results between medical officers and interns

**Figure 2:**
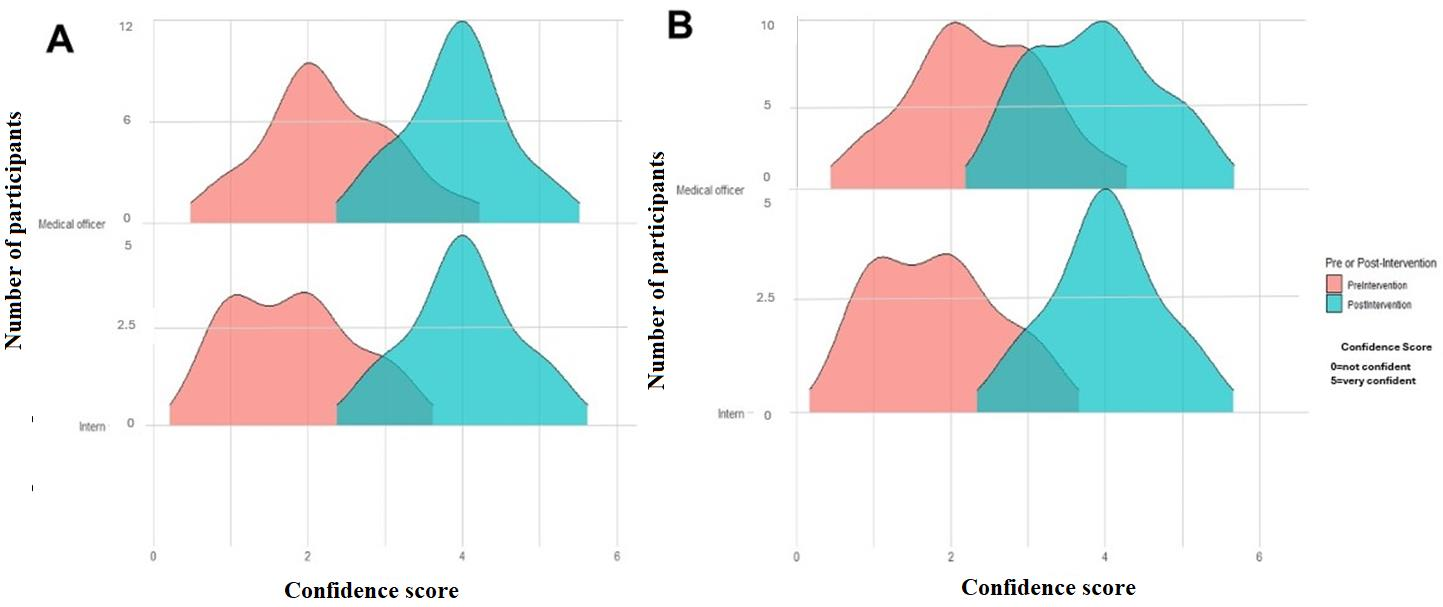
A,B) comparison of participant´s confidence in diagnosing and treating common dermatological conditions pre and post intervention

**Figure 3:**
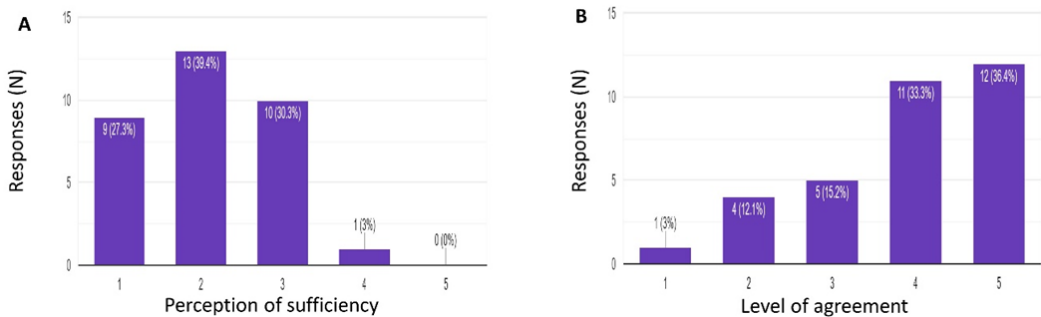
A,B) perceptions of medical doctors on the sufficiency of dermatology training at a undergraduate level and their level of agreement in expanding undergraduate dermatology teaching

**Outcome data and main results:** the pre-intervention test score was 67.11% in comparison to the post-intervention mean score of 92.50%. The difference between means (post-intervention - pre-intervention) ± SEM was 25.39 ± 4.81 with a p-value of <0.0001. The breakdown between career levels showed the Intern´s median pre-intervention score of 60% versus the medical officer´s median pre-intervention score of 70%. Both the intern´s and medical officer´s post-intervention median score was 95% (Figure 1). Overall, the confidence levels of both interns and medical officers in diagnosing and treating common dermatological conditions improved post-intervention in comparison to pre-intervention (Figure 2(A,B)). The majority of participants found their undergraduate training insufficient in preparing them to manage dermatology conditions post-graduation (Figure 3A) and felt that more time should be dedicated to undergraduate dermatology training (Figure 3B).

## Discussion

This study aimed to look at the baseline dermatological knowledge of medical doctors and determine the effect of fundamental dermatology education on these doctors in improving their level of knowledge on common dermatological conditions. Our study showed that there is a low level of confidence amongst these medical doctors in diagnosing and treating common dermatological conditions which ultimately results in a high referral rate to dermatologists and tertiary hospitals leading to detrimental effects on an already constrained health-care system. This study also highlights the fact that many medical doctors are not confident in managing dermatological conditions and it is important to recognise this in order to make improvements within our healthcare education system.

Skin and subcutaneous disorders were found to be the 4^th^ leading cause of non-fatal disease globally in the years 2010 and 2013 [4,5]. Studies within South Africa have characterized dermatological conditions as a causative factor for a significantly poorer quality of life even when compared to other severe debilitating conditions such as depression and anxiety [5]. In 2017, it was found that there were 220 dermatologists practicing in South Africa with a ratio of one dermatologist to 216000 people [6]. If medical doctors could identify and treat common skin conditions this burden on dermatologists would decrease.

An issue that arises from poor dermatology training amongst doctors is the high frequency of emergency department misdiagnoses, which could result in the administration of unnecessary medications and extra costs [1]. When misdiagnosis leads to inappropriate antibiotic prescription, not only do healthcare costs increase but inappropriate antibiotic use can worsen the emergence of antibiotic-resistant pathogens [7]. Emergency department misdiagnoses also result in an increased burden on dermatologists [1]. Inaccurate diagnosis and management of these conditions can have negative effects on patients as well as the healthcare system and so it is important to identify and address these issues.

The majority of participants in this study felt that their undergraduate training was insufficient in preparing them for the management of common dermatological conditions and felt that more time should be dedicated to dermatology training in the undergraduate medical program. This is an important finding because it is helpful in explaining why medical doctors lack confidence in managing skin disease and provides us with evidence that more time needs to be dedicated towards dermatology. In another study that aimed to explore extended dermatology time for medical students it was found that on average, about 10 hours in 4 years was devoted to dermatology training [8], and with dermatological conditions being extremely prevalent, these shortfalls in medical training should be addressed. Our results also show that medical officers performed better than Interns on the pre-intervention test which is an interesting finding because it would be expected that interns, being recent graduates of medical school, would have retained more knowledge from their more recent undergraduate training. This again emphasizes the need for improved undergraduate dermatology teaching. In this study, intern scores improved slightly more than medical officer scores post-intervention, indicating a catch-up in knowledge and proving the usefulness of such interventions.

The overall pre-intervention results show that medical doctors' baseline knowledge of dermatological conditions is fairly poor. Such educational interventions as implemented in our study have a significant potential in improving the level of knowledge amongst doctors in both diagnosing and treating these conditions. This could ultimately improve the management of patients at a primary care level and reduce the pressure on specialists in treating conditions that should be managed at a primary care level, thus giving dermatologists more time to manage more complex conditions. The World Health Organisation (WHO) also recently outlined the need for a greater emphasis on training of health practitioners at both the primary care and specialist levels in identifying and managing dermatologic conditions [9]. Our study proves that similar interventions could be implemented to align with the WHO goals.

It is crucial to acknowledge the limitations of this study. While conducting the post-test immediately after the intervention helps eliminate external influences like exposure to additional information, it restricts the examination to only the short-term effects of the intervention, without assessing the long-term retention of knowledge. Additionally, opting for an online platform, while preferable for COVID-19 precautions, may constrain interaction and hinder the ability to gauge the participants' level of engagement.

## Conclusion

This study underscores the pressing need for improved dermatological education among medical doctors. The low baseline knowledge and confidence levels observed among participants highlight a systemic issue that contributes to high referral rates to specialists and tertiary hospitals, straining healthcare systems already facing significant challenges. Addressing this deficiency in undergraduate training is imperative to equip doctors with the skills necessary to diagnose and manage common dermatological conditions effectively. Moreover, the findings emphasize the potential of educational interventions to bridge knowledge gaps and enhance patient care at the primary care level, thus alleviating the burden on specialists. Aligning with the World Health Organization's recommendations, interventions aimed at enhancing dermatological training among healthcare practitioners hold promise for mitigating the adverse effects of insufficient dermatological care on patients and healthcare systems.

### What is known about this topic

- *Dermatological conditions are amongst the most frequent complaints affecting communities, it is therefore important for dermatological conditions to be appropriately identified and managed;*
- *Dermatology has become an overlooked aspect of the medical school curricula;*
- *There is a need for greater emphasis in training of health practitioners at both the primary care and specialist levels in identifying and managing dermatologic conditions*.

### What this study adds

- *Many medical doctors lack confidence in diagnosing and treating common dermatological conditions and believe that undergraduate training didn´t adequately prepare them for managing such cases;*
- *Similar educational interventions may be useful in improving the diagnosis and management of dermatological conditions;*
- *Our results are thought-provoking and pave the way for further research in the arena of medical education around dermatological diseases*.
